# Carotid artery stenting in a single center, single operator, single type of device and 15 years of follow-up

**DOI:** 10.1186/s42155-018-0008-2

**Published:** 2018-07-17

**Authors:** Victoria Mayoral Campos, José Andrés Guirola Órtiz, Carlos Tejero Juste, María José Gimeno Peribáñez, Carolina Serrano, Cristina Pérez Lázaro, Ignacio de Blas Giral, Miguel Ángel de Gregorio Ariza

**Affiliations:** 1 GITMI (Grupo de Investigación en Tecnicas de Minima Invasión) del Gobierno de Aragon, Zaragoza, Spain; 2 Servicio de Radiología intervencionista, Hospital Clínico Universitario Lozano Blesa, Avenida San Juan Bosco 15, 50009 Zaragoza, Spain; 3 Servicio de Neurología, Hospital Clínico Universitario Lozano Blesa, Zaragoza, Spain; 4 Departamento de Patología Animal, Facultad de Veterinaria, Zaragoza, Spain

**Keywords:** Neurointervention, Endovascular treatment, Carotid stenting, Revascularization, Carotid artery, Carotid artery stenosis, Stroke, Long-term follow-up

## Abstract

**Background:**

Revascularization with carotid stent (CAS) is considered the therapeutic alternative to endarterectomy (CEA). However, its role compared to CEA remains questioned, mainly due of the heterogeneity of long-term results. The objective of this study was to report the efficacy and durability of CAS in terms of stroke prevention in a “real world experience”.

**Method:**

This was a single-center retrospective analysis of 344 patients treated with CAS between January 2001 and December 2015.

The primary outcome of the trial was stroke, myocardial infarction, or death during a periprocedural period or any stroke event over a 15-year follow-up. The secondary aim was to identify risk factors for 30-day complications, long-term neurological complications, and intra-stent restenosis.

**Results:**

The primary composite end point (any stroke, myocardial infarction, or death during the periprocedural period) was 2.3%. The use of an EPD was protective against major complications.

Long-term follow-up was achieved in 294 patients (85,5%) with a median of 50 months (range 0-155 months). Fifty-six (16,3%) died within this period, most commonly of nonvascular causes (4 patients had stroke-related deaths). During the follow-up period, 8 strokes and 3 TIAs were diagnosed (3.2%).

ISR determined by sequential ultrasound was assessed in 4.4% of the patients and remained asymptomatic in all but 2 patients (0.6%). All patients with restenosis underwent revascularization with balloon angioplasty.

**Conclusion:**

The long-term follow-up results of our study validate CAS as a safe and durable procedure with which to prevent ipsilateral stroke, with an acceptable rate of restenosis, recurrence and mortality.

## Background

Cerebrovascular disease is an increasing global health problem, responsible for 10% of deaths, worldwide (Dorn et al., 2012). Carotid artery stenosis due to atherosclerotic disease is liable for approximately 20–30% of these strokes (Chaturvedi et al., 2005; Roger et al., 2011), with this stroke subtype associated with the highest rate of recurrence (Coutts et al., 2008).

The American Heart Association/American Stroke Association (AHA/ASA) (Kernan et al., 2014) considers revascularization with stent to be a therapeutic alternative to surgery to prevent stroke, in select patients with symptomatic carotid artery stenosis, although in many centers surgery is still considered as the gold standard. While this recommendation is supported by multiple large randomized clinical trials, including CREST (Bonati et al., 2015; Brott et al., 2016), we currently lack long-term outcome data for CAS procedures. Further, critics of CAS cite that this approach fails to remove the plaque, which can result in restenosis and potentially new stroke events. With an aging population and an increasing life expectancy, long-term outcomes following CAS are now needed to guide treatment appropriately.

The objective of this study was to report the efficacy and durability of CAS in terms of stroke prevention. The primary outcome of the trial was stroke, myocardial infarction (MI), or death during a periprocedural period (30 days after treatment), or any stroke event over a 15-year follow-up. The secondary aim was to identify risk factors for 30-day complications, long-term neurological complications, and intra-stent restenosis (ISR).

## Methods

This paper has been approved by the Ethics Committee in our Hospital.

### Patients

An observational retrospective study was performed using material retrieved from a database describing a single-center patient cohort. The study included 344 patients treated with CAS between January 2001 and December 2015. All patients had carotid artery stenosis documented by duplex ultrasound (toshiba aplio 300) and confirmed by angiography. In those cases in which the diagnosis was doubtful or unreliable, CT-angiography (36,3%) or MRI-angiography (45,3%) was performed.

Inclusion criteria were: age > 18 years, with no upper limit; symptomatic stenosis > 50%; asymptomatic stenosis > 60%, with more than one risk factor for future embolism (i.e. progressive carotid stenosis, silent stroke documented by neuroimaging, contralateral carotid occlusion with high-risk carotid plaque, or microemboli detected by transcranial duplex ultrasound).

Exclusion criteria included: life expectancy < 1 year; intracranial hemorrhage or major surgery within 30 days of the procedure; uncontrolled arterial hypertension or coagulopathy; contraindications to heparin or antiplatelet therapy; a lack of percutaneous vascular access.

### Pretreatment evaluation

The human group that worked as a team in all the procedures was formed by neurologists and interventional radiologists, with whom an anesthetist collaborated. The team performs a joint assessment of each case to determine the most appropriate treatment for each patient.

Pretreatment evaluation included an assessment of the degree of stenosis using noninvasive imaging, a neurological assessment (NIH stroke scale), laboratory results, and a 12-lead ECG. Any changes in medication were agreed by the medical team.

All patients agreed to, and provided, written (signed) informed consent. Patients received antiplatelet therapy with daily oral entericoated aspirin (100 mg per day) and clopidogrel (75 mg per day) at least 3 days prior to the procedure. Patients using long-term anticoagulation had their treatment converted to heparin.

### Procedure protocol

CAS procedures were performed by 2 interventional radiologists, one with more than 30 years of experience in endovascular techniques. In most patients, and when practicable in the hospital setting, endovascular treatments were performed within the first 2 weeks of becoming symptomatic (76,4%), except in those cases where there was a high risk of bleeding and hyperperfusion syndrome (Kernan et al., 2014; Furie et al., 2011; Sacco et al., 2006).

The right common femoral artery (CFA) is the preferred access for CAS. The left CFA and the brachial artery were alternative accesses if the right CFA was not possible. A 6 French sheath and a 0.035″ hydrophilic wire (Terumo Europe) is advanced into the aorta under direct fluoroscopy. A multiside-hole pigtail catheher (Cook Medical, Bloomington, IN) was placed over the guide wire and positioned in the aortic arch. An aortogram was obtained with 30 cm^3^ of contrast at 15 ml/s (Ioversol 320 mg/ml).

Catheter selection for CCA was chosen depending on the aortic arch anatomy. The used catheter was a 5 Fr Vertebral (Terumo, Europe). Selection of great vessels in the setting of Type III arch typically required a reverse curve catheter type Simmons II (Terumo Europe) or a brachial approach when femoral access was not possible. A carotid angiography was performed in AP, lateral, and intra-cerebral (Towne and lateral views).

A 260 cm safety “J” guidewire was advanced to the ECA, and a 6 Fr - 90 cm guiding catheter (Flexor - Cook Medical, Bloomington, IN) was then placed in the CCA. Anticoagulation was infused with a bolus of 80 IU/kg of UFH. In scenarios of appropriate anatomic conditions an embolic protection device (EPD; Accunet, Abbott Vascular, Santa Clara, CA) was deployed distally (4.5–6.5 mm). The authors tried to use EPD in all patients regardless of the type of plaque. A self-expanding carotid stent system (Acculink, Abbott Vascular, Santa Clara, CA) was then placed across the stenosis.

Predilation of the stenosis after the placement of the EPD and before stent deployment was controversial. The IR’s performed predilatation when the stent cannot be safely advanced. If predilation was desired, a 5 × 20 mm diameter balloon should suffice and atropine was given prophylactically if bradycardia ensues (procedure was perform always with an anesthesiologist).

A repeat arteriogram was performed. Any residual stenosis exceeding 30% was treated with a 5 × 20 mm diameter balloon angioplasty and atropine was given prophylactically. Two antiplatelet agents, clopidogrel (75 mg for 4 to 6 weeks), and aspirin (100 mg; used indefinitely),

### Data collection and follow-up

We retrospectively collected all clinical, angiographic, and procedural data. A neurological specialist performed the clinical follow-ups, with carotid ultrasound at 1, 3, 6, and 12 months after the intervention, and annually thereafter. If there was any alteration in carotid ultrasound (PSV > 200 or ICA/CCA ratio > 3), a selective angiogram was performed.

All patients were treated with BMT. We made strict control of blood pressure to keep levels < 120/80, LDL cholesterol to keep levels < 70 and blood sugar control to keep levels of HbA1c < 7%. We make patients aware of a healthy lifestyle attempting to be physically active, non-smokers and trying to maintain a healthy body weight.

### Definitions

Patients were considered symptomatic if they presented with ipsilateral amaurosis fugax, transient ischemic attack (TIA), or ischemic stroke within 4 months of the procedure. Stroke was defined as a neurological deficit of cerebrovascular cause that persists beyond 24 h, or a new cerebrovascular lesion in neuroimaging. TIA was defined as a focal neurologic deficit that resolves completely within 24 h. A MI component was defined on the basis of elevated myocardial enzymes plus either symptoms or electrocardiographic evidence of an event. Technical success was defined as restoration of cerebral flow through the lesion, with a > 20% improvement in stenosis and a residual stenosis of < 50%. Major complications included death, stroke or MI. Minor complications were defined as all those that do not require any intervention. Global complications were defined as the sum of major and minor complications. Restenosis was classified when the intra-stent stenosis was greater than 50% (Higashida et al., 2009). Efficacy was defined as the absence of stroke during follow-up. Neurological death was defined as that caused by stroke or associated with procedural complications. Vascular death was defined as that caused by MI or peripheral artery disease (PAD).

### Aims and statistical analysis

The primary aim of the study was to analyze stroke, MI, or death during the periprocedural period (30 days after treatment), or any stroke-event over a 15-year follow-up. We also evaluated the long-term behavior of the implanted carotid stents in terms of patency rate and the need for reintervention. Our secondary aim was to identify predictive risk factors for 30-day complications, long-term neurological complications, and ISR.

Statistical analyses were with the SPSS software (Released 2012; IBM SPSS Statistics for Macintosh; Version 21.0. Armonk, NY: IBM Corp). Normality was tested using the Kolmogorov-Smirnoff test. Quantitative variables were expressed as mean values with standard deviations. Qualitative data were expressed as the total number of events with percentages. Continuous variables were analyzed using either the Student’s t-test or the Mann-Whitney U test. The Chi-squared and Fisher’s exact tests, or likelihood ratios, were used for categorical variables. Survival curves were calculated using the Kaplan-Meier method and were compared with the log-rank test. All tests were considered statistically significant if the *p* value was less than 0.05.

## Results

From January 2001 through December 2015, a total of 344 patients with a median age of 70.79 years (range 44–86) were treated with CAS. The procedure was more common among men than women, with 86% of patients male. All but 35 (10.2%) patients were symptomatic. Demographics, risk factors, and patient morbidities are listed in Table 1. Hypertension, dyslipidemia, and smoking were the most potent, prevalent, and treatable risk factors for stroke.

**Table 1:** Baseline characteristics of patients

Vascular risk factors	Men (*n* = 296)	Women (*n* = 48)	Total (*n* = 344)	p
Hypertension	69,6%	70,8%	69,8%	0,862 ^X2^
PAD	27,7%	16,7%	26,2%	0,107 ^x2^
Previous TIA	7,1%	4,2%	6,7%	0,754 ^F^
Previous Stroke	10,1%	8,3%	9,9%	> 0,999 ^F^
Dyslipidemia	59,1%	64,6%	59,9%	0,474 ^x2^
Smoking	41,6%	10,4%	37,2%	< 0,001 ^x2^
Alcohol	3,4%	0,0%	2,9%	0,368 ^F^
DM	33,8%	25,0%	32,6%	0,228 ^x2^
Obesity	18,9%	29,2%	20,3%	0,102^x2^
Arrhythmias	9,1%	18,8%	10,5%	0,043 ^x2^
Ischemic heart disease	26,0%	14,6%	24,4%	0,087 ^x2^
Cervical radiotherapy	2,4%	2,1%	2,3%	> 0,999 ^F^

X2: Chi-squared test; F: Fisher’s exact tests

The carotid stent deployed was the Acculink with no procedures aborted before insertion of the stent. Protection devices were used for 60.2% of patients. Post-stent balloon dilatation was performed in 73.8% of patients, with predilatation necessary for 20/344 arteries (5.8%).

Technical success was achieved in 335 of the 344 arteries (97.4%) treated. Although it was possible to pass a wire through the stenosis in 9 patients, complete stent expansion was not achieved for this group.

The primary composite end point was 2.3% (Table 2). Strokes occurred in 8 patients, with the only death related to severe bleeding in the context of a hyperperfusion syndrome. None of the stroke events were the result of an acute stent thrombosis. There were no patients with MI.

**Table 2:** Peri-procedural complications

Complications	n	%
Majors	8	2,3%
MI	0	0,0%
Stroke	7	2,0%
Fatal Stroke (death)	1	0,3%

We undertook exploratory analyses of the composite outcome of stroke, death, or procedural myocardial infarction. These analyses suggested that use of an EPD was protective against major complications (Table 3). In addition, EPD-use led to a reduction in major complications, falling from 5,1% without the use of EPDs to 0.5% with EPDs (*p* = 0.005).

**Table 3:** The influence of procedural characteristics on peri-procedural complications

Variable	Major complicatons				Minor complications			
		n	%	p		n	%	p
Ulcer	No	8	3,3%	0,111^F^	No	96	39,7%	0,109^x2^
	Yes	0	0,0%		Yes	50	49,0%	
Pre-stent angioplasty	No	8	2,5%	> 0,999^F^	No	131	40,4%	0,002^x2^
	Yes	0	0,0%		Yes	15	75,0%	
EPD	No	7	5,1%	0,005^X2^	No	46	33,6%	0,007^x2^
	Yes	1	0,5%		Yes	100	48,3%	
Post-stent angioplasty	No	2	2,2%	> 0,999^F^	No	34	37,8%	0,297^x2^
	Yes	6	2,4%		Yes	112	44,1%	

X2: Chi-squared test; F: Fisher’s exact tests

**Fig. 1:**
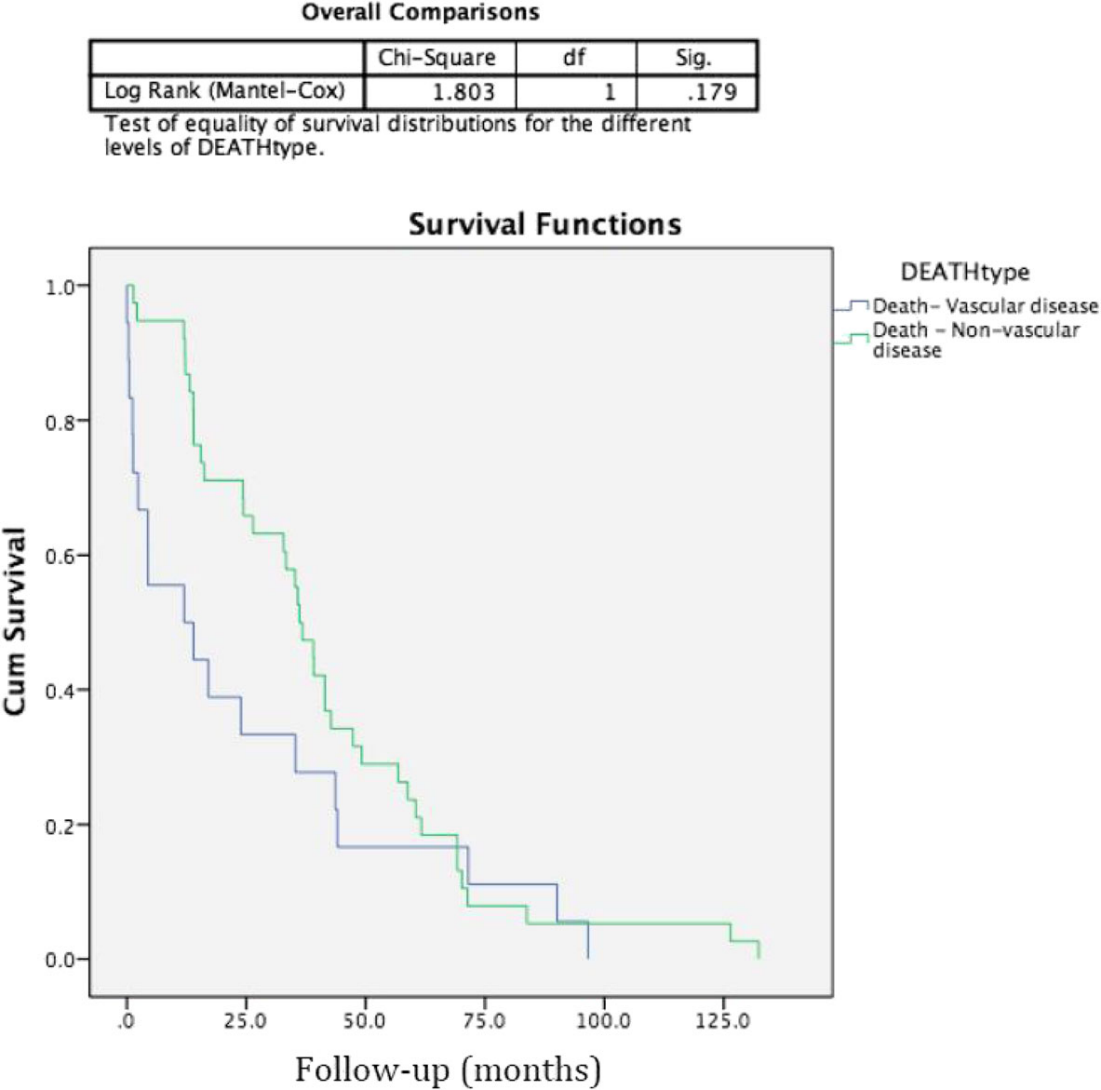
Survival curves for vascular and non vascular deaths

For long-term analysis, patients were followed for a median of 50 months (range 0-155 months). A total of 50 patients were lost to follow-up (Table 4), with long-term follow-up achieved in 294 patients (85.5%). Fifty-six (16.3%) patients died within this period, including 4 stroke-related deaths, and 52 non-lesion related deaths due to comorbidity. Two patients died from intraparenchymal hemorrhage and two from ischemic stroke, one ipsilateral to the treated carotid artery and the other, contralateral to the treated carotid artery. Among the non-neurological deaths, there were 5 cases of vascular death: 3 cases due to chronic ischemia of the lower extremities, and 2 due to acute myocardial infarction (Fig 1).

**Table 4:** Follow-up period

Follow-up (months)	n	%	Average	SD	Median	(IQR)
Keep in follow-up	238	69,19%	56,40	36,30	48,90	(59,50)
Loss to follow-up	50	14,53%	34,08	29,28	30,04	(41,50)
Neurological death	4	1,16%	10,36	10,69	8,14	(19,25)
No neurological death	52	15,12%	38,33	31,28	35,53	(44,50)

During the follow-up period, 8 strokes and 3 TIAs were diagnosed (3.2%). The majority of the strokes (75%) were contralateral to the treated carotid artery and most were ischemic. We did not identify any predictive risk factors or any procedural characteristics for ipsilateral neurological complication.

ISR was assessed in 4.4% of the patients and remained asymptomatic in all but 2 patients (0.6%). All patients with restenosis underwent revascularization with balloon angioplasty. No stent fracture was observed. Pre-stent angioplasty was significantly associated with the incidence of ISR (Table 5). Risk factors were neither related to neurological complications nor ISR. Further, there were significant differences between the degree of restenosis and the time of its appearance: the greater the degree of restenosis, the shorter the post-procedure time period (*r* = − 0.426; *p* = 0.038). We found no association between restenosis and recurrent events.

**Table 5:** The influence of procedural characteristics on restenosis

Proceure	Reestenosis	n	%	p
Peri-procedural complications	No	142	6,8%	0,866^x2^
	Yes	152	7,2%	
Pre-stent angioplasty	No	322	5,6%	0,001^LR^
	Yes	20	25,0%	
Post-stent angioplasty	No	77	9,1%	0,353^LR^
	Yes	254	6,3%	

X2: Chi-squared test; LR: likelihood ratios

## Discussion

Stroke is a major cause of mortality and morbidity in industrialized countries. These data, combined with an ever-increasing life expectancy, necessitates that we conduct more detailed analyses of whether carotid revascularization is a safe and effective treatment with which to prevent stroke.

In our series, the 30-day outcomes after CAS show that carotid stenting is an effective treatment in preventing future vascular events, with a low incidence of periprocedural complications: 2.3% for disabling stroke, death, or MI. Previous systematic reviews of nonrandomized cases series (Kastrup et al., 2003), as well as several studies (Castriota et al., 2002; Cremonesi et al., 2003; Garg et al., 2009; Giri et al., 2015), have shown that EPD-use appears to reduce the incidence of new ischemic lesions. However, various reports have also criticized the use of EPDs in CAS as these devices must pass through the arterial stenosis, which might itself provoke complications (Reimers et al., 2004; Wu et al., 2011) and a greater incidence of microemboli (El-Koussy et al., 2007). Nevertheless, the results of our series show a decreased rate of major complications when EPDs are used (*p* = 0.005), which therefore classifies these devices as protective against stroke.

To our knowledge, our study comprises the largest patient cohort, with the longest national follow-up to compare stroke prevention in patients treated by the same surgical team. In our series, stenting was performed electively as an alternative to endarterectomy. This is because the committee composed by neurologist, radiologist, and interventional radiologist (vascular surgeons refused to participate) decided, more than 15 years ago to send patients directly to CAS, depending on the poor results of the surgery department. Thus, our indications were not limited to patients at a high surgical risk. Nevertheless, the risk of periprocedural complication was low, and similar to data from surgical registries with more stringent inclusion criteria (North American Symptomatic Carotid Endoarterectomy Trial Collaboration, 1991; Executive Committee for the Asymptomatic Carotid Atherosclerosis Study, 1995).

The CREST study showed no significant differences between stent and endarterectomy in terms of the risk of stroke or death over its 10-year follow-up. Indeed, the higher risk of stroke reported for CAS can be attributed to the periprocedural differences between the two groups (Brott et al., 2016; Brott et al., 2010), and the infrequent use of EPDs (El-Koussy et al., 2007). Other randomized trials comparing both techniques have been reported, and showed no significant differences after long-term follow-up (Bonati et al., 2015; Gurm et al., 2008; Mas et al., 2008).

Our data appears to indicate that CAS is preventive against ipsilateral stroke with a low long-term risk of severe stroke The recurrence rate in our series of 3.2% primarily involved strokes contralateral (75%) to the treated carotid artery, and most were ischemic. This finding has also been reported in previous related studies in which no significant difference in cumulative rates of fatal or disabling stroke were found for stenting vs. endarterectomy (Bonati et al., 2015; Mas et al., 2008). It is interesting to note that our observed stroke rate of 3.2% is (fairly) consistent with, and even lower than the 8.5% reported in the ECST at 3 years (European Carotid Surgery Trial, 1998), and the 13% rate of the NASCET (North American Symptomatic Carotid Endoarterectomy Trial Collaboration, 1991). However, our patient population was closely monitored in terms of risk factors, which may have lowered our rate of complication. Regarding risk factors, Donato et al. (de Donato et al., 2008) and Brooks et al. (Brooks et al., 2014) found that being symptomatic at the time of enrollment and intervention was a good predictor of early and late neurological complication. We failed to find any risk factor associated with late neurological complications, although this may be related to our low recurrence rate.

Prevention of ipsilateral stroke is the ultimate goal of any treatment for carotid stenosis. However, durability, defined as patency determined by Doppler ultrasound, may also serve as a useful definition of therapeutic utility. Our analysis of long-term outcomes showed that the long-term rate of restenosis after stenting was low (4.4%), and comparable, or even lower, than those achieved with CEA. However, direct comparisons are complicated by the criteria used to make a diagnosis in Doppler sonography. Of the restenosis cases, only 2 (0.6%) were symptomatic. We found no association between restenosis and recurrent events, but pre-stent angioplasty was significantly associated with the incidence of ISR. This is difficult to interpret given the small number of recurrent events, pre-stent angioplasty, and restenoses seen in our study. A certain amount of controversy surrounds the issue of long-term rates of restenosis after stenting vs. endarterectomy. Some trials (Bonati et al., 2015; Brott et al., 2016; Mas et al., 2008) have demonstrated no differences when comparing both techniques. For example, 5-year risks of 10.8% vs. 8.6%, 3-year risks of 3.3% vs. 2.8%, and 2-year risks of 6.0% vs. 6.3%, for stenting vs. endarterectomy, have been reported, respectively. In contrast, the CAVATAS (Bonati et al., 2009) and SPACE trials (Eckstein et al., 2008) reported higher rates of restenosis 2 years after treatment in the stenting group vs. endarterectomy group (10.7% vs. 4.6%). Consequently, further studies are needed to compare the rate of restenosis and to investigate the association between restenosis and recurrent stroke.

The current study has several limitations. The main limitation was its small sample size and retrospective design. Moreover, results obtained in this study were compared with the literature, as it was not possible to undertake a randomized comparison of CAS versus CEA. Further, the efficacy of the EPDs could not be evaluated throughout the entirety of this study as these devices were not available until 2005.

## Conclusion

The long-term follow-up results of our study validate CAS as a safe and durable procedure with which to prevent ipsilateral stroke. Our data also suggest that CAS is effective in terms of long-term functional outcome and risk of fatal or disabling stroke. Post CAS restenosis was infrequent, and, in the majority of cases, asymptomatic.

## Abbreviations

CAS: Carotid artery stent
CCA: Common carotid artery
CEA: Carotid artery endarterectomy
CFA: Common femoral artery
CT: Computerized axial tomography scan
ECA: External carotid artery
EPD: Embolic protection device
IRs: Interventional radiologists
ISR: Intra-stent restenosis
MI: Myocardial infarction
MRI scans: Magnetic resonance imaging
PAD: Peripheral artery disease
PSV: Peak systolic velocity
TIA: Transient ischemic attack
